# More than one fungus in the pepper pot: Integrative taxonomy unmasks hidden species within *Myriostoma coliforme* (Geastraceae, Basidiomycota)

**DOI:** 10.1371/journal.pone.0177873

**Published:** 2017-06-07

**Authors:** Julieth O. Sousa, Laura M. Suz, Miguel A. García, Donis S. Alfredo, Luana M. Conrado, Paulo Marinho, A. Martyn Ainsworth, Iuri G. Baseia, María P. Martín

**Affiliations:** 1 Programa de Pós-Graduação em Sistemática e Evolução, Universidade Federal do Rio Grande do Norte, Natal, Rio Grande do Norte, Brazil; 2 Jodrell Laboratory, Royal Botanic Gardens Kew, Richmond, Surrey, England; 3 Department of Biology, University of Toronto, Mississagua, Ontario, Canada; 4 Graduação em Ciências Biológicas, Universidade Federal do Rio Grande do Norte, Natal, Rio Grande do Norte, Brazil; 5 Departamento de Biologia Celular e Genética, Universidade Federal do Rio Grande do Norte, Natal, Rio Grande do Norte, Brazil; 6 Departamento de Botânica e Zoologia, Universidade Federal do Rio Grande do Norte, Natal, Rio Grande do Norte, Brazil; 7 Departamento de Micología, Real Jardín Botánico-CSIC, Plaza de Murillo 2, Madrid, Spain; Brigham Young University, UNITED STATES

## Abstract

Since the nineteenth century, *Myriostoma* has been regarded as a monotypic genus with a widespread distribution in north temperate and subtropical regions. However, on the basis of morphological characters and phylogenetic evidence of DNA sequences of the internal transcribed spacer (ITS) regions and the large subunit nuclear ribosomal RNA gene (LSU), four species are now delimited: *M*. *areolatum* comb. & stat. nov., *M*. *calongei* sp. nov., *M*. *capillisporum* comb. & stat. nov., and *M*. *coliforme*. *Myriostoma coliforme* is typified by selecting a lectotype (iconotype) and a modern sequenced collection as an epitype. The four species can be discriminated by a combination of morphological characters, such as stomatal form, endoperidial surface texture, and basidiospore size and ornamentation.

## Introduction

Correct species recognition is an essential requirement for the understanding of systematics, evolution and ecology. Furthermore, it is a prerequisite for population biological studies, reliable Red List assessments and effective conservation action. Recent molecular studies suggest that the magnitude of fungal taxonomic diversity is seriously underestimated [1–3]. Basidiomycete taxonomy has been revolutionized by the use of molecular techniques, which have been particularly valuable in revealing component cryptic or semi-cryptic taxa within species complexes or aggregates [4–6]. The drawbacks associated with the traditional morphology-only approach are succinctly expressed by Stielow et al. [7]: “The difficulties in defining characters and their states, and particularly the fact that distinct taxonomists assigned distinct weights to morphological characters, have probably been the largest obstacles to the establishment of broadly acceptable classifications of numerous difficult groups of fungi”.

Historically, the gasteroid genera *Astraeus* Morgan and *Pisolithus* Alb. & Schwein. (Boletales) have been regarded as monotypic. However, recent molecular analyses, mainly of the universally adopted DNA barcode region for fungi [8], the internal transcribed spacer region of nuclear ribosomal DNA (ITS), have revealed the presence of previously hidden taxa. Several species have now been described within these genera and several more await formal naming and description [9–12].

The genus *Myriostoma* Desv., typified by *Myriostoma anglicum* Desv. (an illegitimate name for *M*. *coliforme*) is a very distinct and rare gasteroid genus of the family Geastraceae (Basidiomycota) which, until now, has been regarded as monotypic. Although *M*. *coliforme* (With.) Corda has been adopted as the correct name by some authors [13,14], the correct authorship for the name is *M*. *coliforme* (Dicks.) Corda. The single species is considered to have a worldwide distribution [13,15–22]. It is popularly known as pepper pot earthstar, and historically as cullender (colander) puffball, and is considered to be easily recognizable by its combination of unique characters, such as multiple endoperidial stomata (pores) and pedicels (stalks) and basidiospores with a wing-like reticulate ornamentation [21,23], characters that are absent in the earthstar genus *Geastrum* Pers. Two varieties have been described based on morphological characters: *M*. *coliforme* var. *capillisporum* V.J. Staněk from Cape Province, South Africa [24], and *M*. *coliforme* var. *areolatum* Calonge & M. Mata from Costa Rica [16].

Although *M*. *coliforme* is considered to be rare across the continent and red-listed in 20 European countries, evidence of large-scale population decline is lacking [22]. Moreover, contributors to the IUCN Global Fungal Red List Initiative (http://iucn.ekoo.se/iucn/species/122233/) indicated that populations are increasing in some European countries and likely to be currently underestimated globally. The main aim of the current study is to apply a combined molecular and morphological (integrative taxonomic) analysis to specimens identified as *M*. *coliforme* to investigate whether the name has been applied to a suite of hidden species as was previously shown to be the case in *Astraeus* and *Pisolithus*.

## Materials and methods

### Morphological studies

The morphological analyses were performed on specimens, including types, deposited in the Fungal Collection of the Federal University of Rio Grande do Norte (UFRN Herbarium), the collection of fungi of the Real Jardín Botánico of Madrid (MA-Fungi), the cryptogamy collection (PC) at the Herbarium of the Muséum national d'Histoire naturelle (MNHN—Paris), and the Fungarium of the Royal Botanic Gardens, Kew (K), (Table 1). Macromorphological studies were based on 26 exsiccates using a Nikon H600L stereomicroscope coupled with a Nikon DS-Ri camera for image capture. Colour descriptions followed Kornerup and Wanscher [25]. For micromorphological features, such as basidiospores, eucapillitium and exoperidial hyphae, a Nikon Eclipse Ni light microscope (LM) coupled with a Nikon DS-Ri camera was used. Basidiospore measurements were made at 1000× magnification following Sousa et al. [21] and include ornamentation. Scanning electron microscopy (SEM) was used to observe the patterns of ornamentation on basidiospores, eucapillitium and endoperidial surfaces.

**Table 1:** Specimens and sequences included in this study.

Species	Country: Locality	Collection year	Fungarium number	GenBank accession number	
				ITS	LSU
***Myriostoma areolatum*** *comb*. *& stat*. *nov*.	Costa Rica: San José	1991	MA-Fungi 36165, paratype	**KY096673**	**KY096690**
	Costa Rica: Guanacaste	2005	MA-Fungi 68596, isotype	-	-
***Myriostoma calongei*** *sp*. *nov*.	Argentina: Colón	2012	MA-Fungi 83759 (as *M*. *coliforme*), paratype	KF988467	KF988348
	Brazil: Pernambuco	2006	UFRN-Fungos 386, paratype	**KY096674**	**KY096691**
	Brazil: Pernambuco	2007	UFRN-Fungos 990, paratype	**KY096675**	**KY096692**
	Brazil: Río Grande do Norte	2012	UFRN-Fungos 2019, holotype	**KY096676**	**KY096693**
	Brazil: Río Grande do Norte	2006	UFRN-Fungos 2020, isotype	**KY096677**	**KY096694**
***Myriostoma capillisporum*** *comb*. *& stat*. *nov*.	South Africa: Grahamstown	1930s	K(M)205482 (as *M*. *coliforme*)	**KY096678**	**KY096695**
	South Africa: Groot River	1930s	K(M)205483 (as *M*. *coliforme*)	**KY096679**	**KY096696**
	South Africa: Cape of Good Hope	pre 1885	K(M)205540 (as *M*. *coliforme*)	**KY096680**	**KY096697**
***Myriostoma coliforme***	Channel Islands: Jersey	1996	K(M)37233	EU784376	**KY096698**
	Channel Islands: Jersey	1999	K(M)61641	**KY096681**	**KY096699**
	UK: England, East Suffolk	2006	K(M)138625, epitype	**-**	**KY096700**
	UK: England, East Suffolk	2010	K(M)166470	**KY096682**	**-**
	UK: England, East Suffolk	2014	K(M)195584	**-**	**KY096701**
	UK: England, West Norfolk	1880	K(M)81165	**KY096683**	**-**
	France: Région de Nay	1964	PC 0723885	**KY096684**	-
	Hungary: Felsolajos	2003	M. Jeppson 8714*	KC582020	KC582020
	Portugal: Leiria	1993	MA-Fungi 31316	**KY096685**	**KY096702**
	Portugal: Madeira Island	2007	MA-Fungi 75818	**KY096686**	**-**
	Russia: Rostov Region	2004	K(M)154620	**KY096687**	**KY096703**
	Spain: Menorca	1998	MA-Fungi 40288	**KY096688**	**-**
	Spain: Jaén	2004	MA-Fungi 60898	**KY096689**	**KY096704**
	Spain: Madrid	-	JC. Zamora 496*	KF988337	KF988466
	USA: Hawai‘i	-	TNS: TKG-GE-50801	JN845203	JN845328
**outgroup**					
*Geastrum saccatum*	Sweden	-	TK950910	KC581968	KC581968
	Sweden	2000	GH000909	KC581969	KC581969

New sequences in bold.

*Personal Fungarium.

### DNA extraction, PCR amplification and sequencing

Genomic DNA was extracted from approximately 10 mg of gleba of mature dry basidiomata. The DNeasy^TM^Plant Mini Kit (Qiagen, Valencia, CA) was used to isolate DNA from UFRN and MA-Fungi specimens, following the manufacturer’s instructions with the following modifications: glebal masses were macerated in 1.5 ml tubes with a micropestle before suspension in lysis buffer and again after overnight incubation at 55–60°C. Both ITS and the 5’–1450–base region of the LSU were analysed using the primer pairs ITS1F/ITS4 [26–27] and LR0R combined with LR7 or LR5 [28–29] respectively. DNA amplifications were carried out using illustra^TM^ PureTaq^TM^ Ready-To-Go^TM^ PCR Beads (Healthcare, Buckinghamshire, UK), adding 1 μl [10 μM] of each primer and 23 μl of isolated DNA [1.5–5.0 ng/μl]. Cycling conditions followed Martín and Winka [30]. PCR products were verified on 1% agarose gels (UtraPureTM Invitrogen), purified using ExoSAP-IT® (USB Corporation, OH, USA) and sequenced bidirectionally in Macrogen Inc. (Seoul, South Korea).

DNA from specimens K(M)138625 and K(M)61641 was extracted using an enzymatic digestion-glass fibre filtration protocol in 96-well plate format with a vacuum-manifold as described in Dentinger et al. [31]. PCR amplifications and sequencing were performed following Dentinger and Suz [32]. DNA from the rest of the specimens from the Kew Fungarium was extracted and ITS and LSU regions amplified using Extract-N-Amp (Sigma, Dorset, UK).

The resulting sequences were edited and the consensus sequence was obtained using Sequencher 5.2.4 (Gene Codes Corp., USA). Preliminary identifications were performed through megablast searches comparing the newly-generated sequences with those in GenBank [33]. Sequences were submitted to GenBank under the accession numbers indicated in Table 1.

### Sequence alignments and phylogenetic analyses

Both ITS and LSU sequences were aligned separately using Se-Al v. 2.0a11 Carbon [34]. To infer phylogenetic relationships among *Myriostoma* specimens, homologous sequences retrieved from the EMBL/GenBank/DDBJ databases were included in the alignment [35]. Since *Geastrum* is the sister genus of *Myriostoma* [36], two sequences of *Geastrum saccatum* Fr. were included as outgroup.

Where ambiguities in the alignment occurred, the alignment generating the fewest potentially informative characters was chosen [37]. Alignment gaps were marked “-”, unresolved nucleotides and unknown sequences were indicated with “N”. Three types of analyses were carried out for ITS and LSU individual alignments and the combined ITS/LSU alignment: maximum parsimony (MP), maximum likelihood (ML), and Bayesian inference. The combined ITS/LSU alignment was submitted to the TreeBASE Number.

In the MP analyses, minimum length Fitch trees were constructed using heuristic searches with tree-bisection-reconnection branch swapping, collapsing branches if maximum length was zero, with the MulTrees option in PAUP*4.0b10 [38], and a default setting to stop the analyses when reaching 100 trees. Gaps were treated as missing data. Nonparametric bootstrap (MPbs) support [39] for each clade, based on 10,000 replicates using the fast stepwise-addition, was tested [40]. The consistency index, CI [41], retention index, RI [42], and rescaled consistency index, RC [42], were obtained. The ML approach was carried out using RAxML [43] in the CIPRES portal (CIPRES Science Gateway v.3.3) assuming a GTR+I+G model as selected by PAUP*4.0b10; MLbs support for each clade, based on 1,000 replicates was tested. The Bayesian analysis [44–45] was performed using MrBayes 3.2 [46], and assuming the general time reversible model [47], including estimation of invariant sites and assuming a discrete gamma distribution with six categories (GTR+I+G), as selected by PAUP*4.0b10. Two independent and simultaneous analyses starting from different random trees were run for 2.000.000 generations with four parallel chains and trees and model scores saved every 100th generation. The default priors in MrBayes were used in the analysis. Every 1.000th generation tree from the two runs was sampled to measure the similarities between them and to determine the level of convergence of the two runs. The potential scale reduction factor (PSRF) was used as a convergence diagnostic and the first 25% of the trees were discarded as burn-in before stationary was reached. The 50% majority-rule consensus tree and the posterior probability (PP) of the nodes were calculated from the remaining trees with MrBayes. A combination of both bootstrap proportion and PP was used to assess the level of confidence for a specific node [2,48]. The phylogenetic trees were visualized using FigTree v. 1.3.1 (http://tree.bio.ed.ac.uk/software/figtree/) and edited with Adobe Illustrator CS3 v. 11.0.2 (Adobe Systems).

Moreover, Kimura-2-parameter (K2P) pairwise distances between ITS sequences were obtained using PAUP*Version 4.0b10, to delimit species following a barcoding approach [8].

### Nomenclature

The electronic version of this article in Portable Document Format (PDF) in a work with an ISSN or ISBN will represent a published work according to the International Code of Nomenclature for algae, fungi, and plants, and hence the new names contained in the electronic publication of a PLOS ONE article are effectively published under that Code from the electronic edition alone, so there is no longer any need to provide printed copies.

In addition, new names contained in this work have been submitted to MycoBank, from where they will be made available to the Global Names Index. The unique MycoBank number can be resolved and the associated information viewed through any standard web browser by appending the MycoBank number contained in this publication to the prefix at http://www.mycobank.org/MB. The online version of this work is archived and available from the following digital repositories: PubMed Central, LOCKSS and Digital-CSIC.

## Results

This study generated 32 new *Myriostoma* sequences (Table 1). The ITS dataset included 24 sequences, 17 generated in this study and seven obtained from EMBL/GenBank/DDBJ databases. The alignment resulted in 613 unambiguously aligned nucleotide positions (496 constant, 40 parsimony-uninformative, and 77 parsimony-informative). The 100 most parsimonious trees gave a length of 129 steps, CI = 0.9535, HI = 0.0465, and RC = 0.9688. The ML tree and the 50% Bayesian majority rule combined consensus tree (not shown) showed essentially the same topology as the parsimony strict consensus tree (not shown). *Myriostoma* sequences were resolved as monophyletic in a highly supported clade (MPbs = 100%, MLbs = 100%, PP = 1.0). The specimen from Costa Rica (MA-Fungi 36165) was sister to the other *Myriostoma* specimens (MPbs = 100%, Mlbs = 100%, PP = 1.0). The rest of the *Myriotoma* sequences clustered in two main groups: sequences from Europe and USA (Hawai‘i) formed a highly supported clade (MPbs = 99%, MLbs = 100%, PP = 1.0), whereas sequences from Argentina, Brazil and South Africa grouped together in two subgroups, one including sequences of South Africa [K(M)205482, K(M)205483 and K(M)205540] and the other one with those of Argentina and Brazil.

The LSU dataset included 21 sequences, 15 generated in this study and six obtained from sequence databases. The alignment resulted in 1391 unambiguously aligned nucleotide positions (1302 constant, 20 parsimony-uninformative, and 69 parsimony-informative). The 100 most parsimonious trees gave a length of 100 steps, CI = 0.9300, HI = 0.0700 and RC = 0.9533. The ML tree and the 50% Bayesian majority rule combined consensus tree (not shown) showed essentially the same topology as the parsimony strict consensus tree (not shown). In the three analyses, the sequences from South Africa [K(M)205482, K(M)205483 and K(M)205540] were sister to the other *Myriostoma* sequences, although this relationship was weakly supported (MPbs = 52%, MLbs = <50%, PP = 0.65). In the parsimony strict consensus tree, the sequence from Costa Rica appeared as the sister group to those from Argentina and Brazil, but this relationship had very low support (MPbs = 61%); moreover, in the ML and Bayesian analyses, the sequence from Costa Rica was the sister group to the clade formed by sequences from Europe and Hawai‘i, a relationship with moderate support (MLbs = 54%, PP = 0.91).

**Fig 1:**
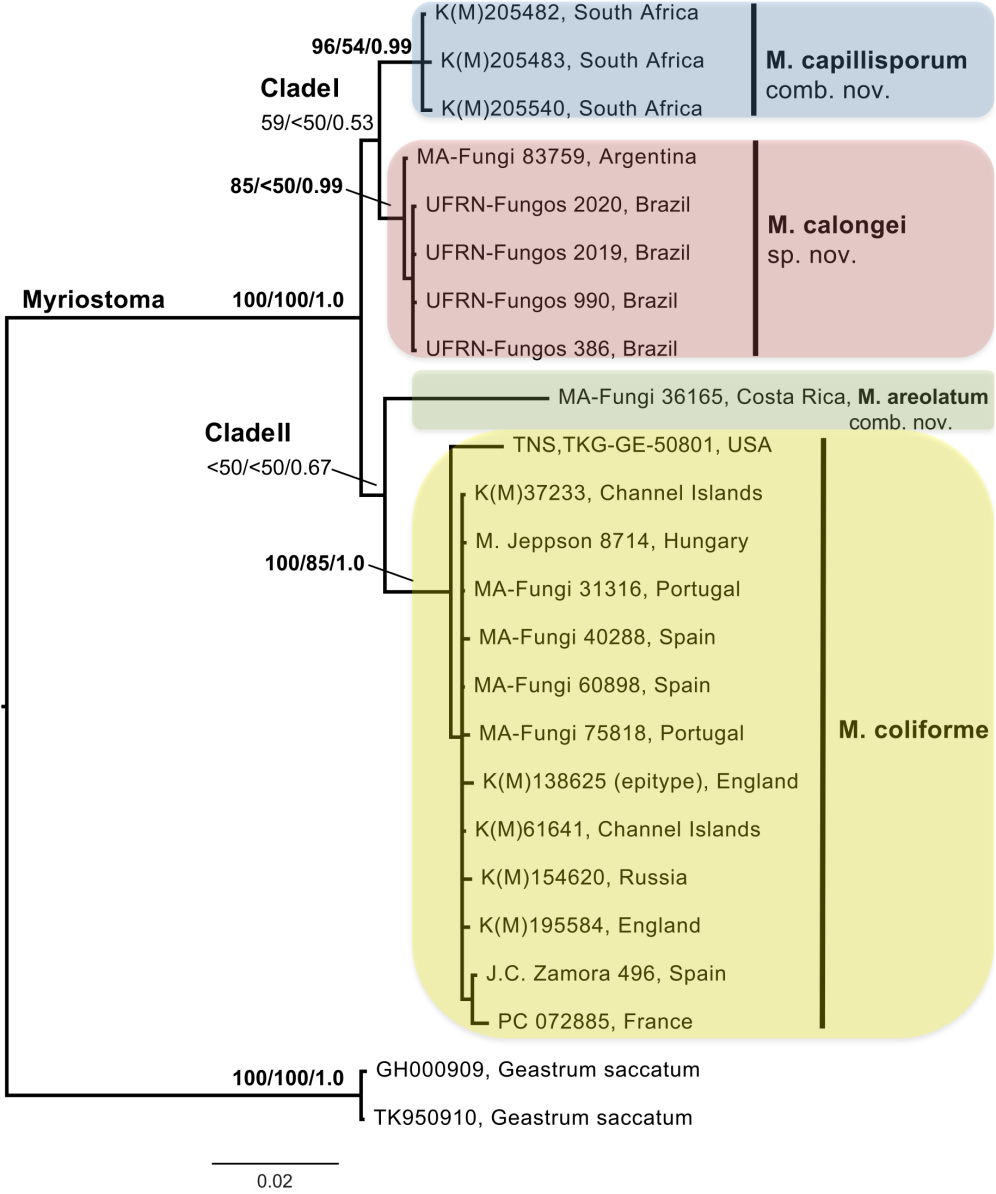
The 50% majority-rule consensus tree of ITS/LSU nrDNA sequences of *Myriostoma* species using a Bayesian approach. Two sequences of *Geastrum saccatum* were used as outgroup. Terminal branches are labelled with appropriate specimen codes and countries of origin. For further specimen details, see Table 1. Numbers at the nodes indicate the percentage bootstrap values obtained from parsimony and maximum likelihood analysis and the posterior probabilities from Bayesian analysis (MPbs/MLbs/PP).

In the ITS/LSU combined dataset there were 2004 unambiguously aligned nucleotide positions (1797 constant, 61 parsimony-uninformative, and 146 parsimony-informative). The 100 most parsimonious trees gave a length of 235 steps, CI = 0.9234, HI = 0,0766, and RC = 0.9474. The ML tree (not shown) and the 50% Bayesian majority rule combined consensus tree (Fig 1) showed essentially the same topology as the parsimony strict consensus tree (not shown). Two main *Myriostoma* clades were produced: Clade I which comprised all sequences obtained from specimens from the Southern Hemisphere, in which the three sequences from South Africa grouped together (MPbs = 96%, MLbs = 54%, PP = 0.99) as did those from Argentina and Brazil (MPbs = 81%, MLbs = <50%, PP = 0.99); and Clade II which comprised all sequences originating from material collected in the Northern Hemisphere, in which all sequences from Europe and the one from Hawai‘i form a highly supported group (MPbs = 100%, MLbs = 85%, PP = 1.0) separated from the Costa Rica sequence.

A revision of the morphological characters present in the analysed material, such as basidiospore size and shape, together with endoperidial surface texture and stomatal morphology, supports the recognition of four distinct species: in Clade I, *M*. *capillisporum* comb. & stat. nov. from South Africa, with basidiospores 7.0–10.9 μm diam with long warts (2.9–6.6 μm high), and a wrinkled to slightly verrucose endoperidial surface; and *M*. *calongei* sp. nov. from Argentina and Brazil, with basidiospores 5.6–8.7 μm, verrucose endoperidium with prominent triangular processes (warts 0.13–0.28 mm high); and in Clade II, *M*. *coliforme*, with basidiospores 6.1–8.0 μm, with a wrinkled to slightly verrucose endoperidial surface (warts < 0.1 mm high), and *M*. *areolatum* comb. & stat. nov. from Costa Rica, with basidiospores 5.6–6.9 μm diam, with a similar endoperidial surface to that of *M*. *coliforme*, and *M*. *capillisporum*.

Furthermore, the K2P pairwise distance of *Myriostoma* ITS sequences included in Table 1 show high genetic variation between the four species considered (Table 2). There are clearly defined barcoding gaps within the ITS sequences of *Myriostoma* such that interspecific variation exceeds intraspecific variation [8]. Based on these results, a new species is described and two varieties are elevated to specific rank. No type material of *M*. *coliforme* is known [13,15] and as Persoon [49] referred to Dickson’s (not Withering’s) name [50], thereby sanctioning it, Dickson’s illustration is selected as lectotype and a recently sequenced collection from the same English region (East Anglia) is designated as epitype (see below).

**Table 2:** Matrix of pairwise Kimura-2-parameter (K2P) distance between ITS sequences from the four species analysed in this paper.

	1	2	3	4
1. *Myriostoma areolatum*	-*			
2. *M*. *capillisporum*	0.06918	**0.00000**		
3. *M*. *calongei*	0.05623	0.01278	**0.00227**	
4. *M*. *coliforme*	0.07711	0.03446	0.05219	**0.00885**

Maximum intraspecific distances in bold; the other values are the minimum interspecific distances.

* The intraspecific value for *M*. *areolatum* is not given in the table, since only one collection was sequenced.

## Taxonomic treatment

### Key to *Myriostoma* species

1 Basidiomata with areolate and tubular stomata …………………………………*M*. *areolatum*
1 Basidiomata with non-areolate and non-tubular stomata …………………………………… 2
2 Endoperidial surface strongly verrucose with warts > 0.1 mm high with (SEM) blunt triangular shape ………………………………………………………………… *M*. *calongei*
2 Endoperidial surface wrinkled to slightly verrucose with warts < 0.1 mm high having (SEM) rounded apices ………………………………………………………………………………… 3
3 Basidiospores (7.0)7.4–10.9 μm diam with prominent ornamentation (2.9–6.6 μm high) comprising (SEM) a reticulum of branching perforated ridges, crests and warts forming arcs and broken circles in face view ……………………………………………………*M*. *capillisporum*
3 Basidiospores 6.1–8.0 μm diam with ornamentation (1.2–1.6 μm high) comprising (SEM) a relatively low simpler reticulum with less curvature in face view ………………………………………………………………………………………*M*. *coliforme*

**Fig 2:**
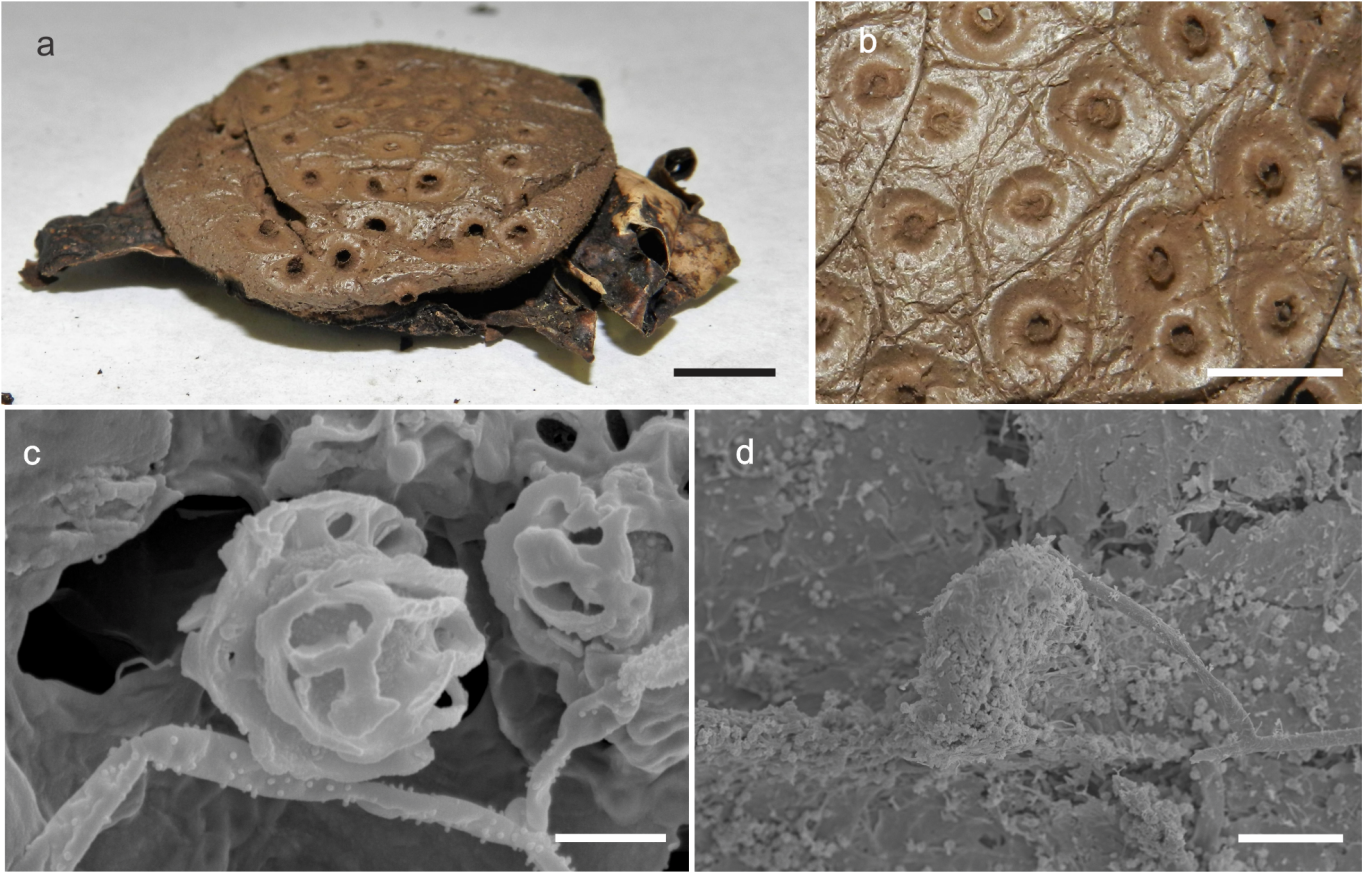
*Myriostoma areolatum* (MA-Fungi 68596, isotype). (a) Dried expanded basidioma *ex situ*, bar = 10 mm. (b) Stomata, bar = 5 mm. (c) Basidiospores under SEM, bar = 2 μm. (d) Endoperidial surface under SEM, bar = 50 μm.

***Myriostoma areolatum*** (Calonge & M. Mata) M.P. Martín, J.O. Sousa & Baseia, comb. & stat. nov.–Fig 2, Mycobank MB 818615

Etymology. Referring to the numerous areolate stomata in the endoperidium.

Basionym. *Myriostoma coliforme* var. *areolatum* Calonge & Mata, *Bol*. *Soc*. *Micol*. *Madrid* 30:116 (2006), MB 546689.

Holotype. Costa Rica, Guanacaste, Parque Nacional Barra Honda, La Capilla, Caverna Pequeña, in soil, 1 Aug. 2005, leg. C. Aguilar 130–05 (USJ 82231!, under *Myriostoma coliforme* var. *areolatum* Calonge & M. Mata).

Diagnosis. *Myriostoma areolatum* can be distinguished from other known *Myriostoma* species by its tubular (up to 1 mm high) and areolate (up to 4 mm diam.) stomata. This species is very close to *M*. *coliforme*, but *M*. *areolatum* has smaller basidiospores (5.6–6.9 μm diam).

Description. Expanded basidiomata arched, 50–80 mm wide. Exoperidium splitting into 7–9 rays, revolute to horizontal, non-hygroscopic. Endoperidial body 35–45 mm wide, shiny, verrucose. Multiple circular stomata (up to 42) of about 4 mm diam, which have an areolate, tubular and fimbriate peristome (1 mm high, 1mm diam). Endoperidial surface ornamentation comprised of small processes with rounded tips (sub SEM). Eucapillitial hyphae brownish, 2.0–5.0 μm diam, surface smooth or with rounded warts, lumen yellowish. Basidiospores globose to subglobose, 5.6–6.9 μm diam, with an ornament of winged ridges 0.8–1.5 μm high; under SEM, the ornamentation is reticulate, comprising warts and branching ridges with planar and curved apices.

Known distribution. Central America (Costa Rica).

Additional specimens examined. Costa Rica, Guanacaste, Parque Nacional Barra Honda, La Capilla, Caverna Pequeña, in soil, 1 Aug. 2005, leg. C. Aguilar 130–05 (MA-Fungi 68596, under *M*. *coliforme* var. *areolatum* Calonge & M. Mata, isotype); San José, Ciudad Colón, Finca “El Rodeo”, 13 Jun. 1991, leg. M.P Núñez (MA-Fungi 36165, under *M*. *coliforme* var. *areolatum* Calonge & M. Mata, paratype).

**Fig 3:**
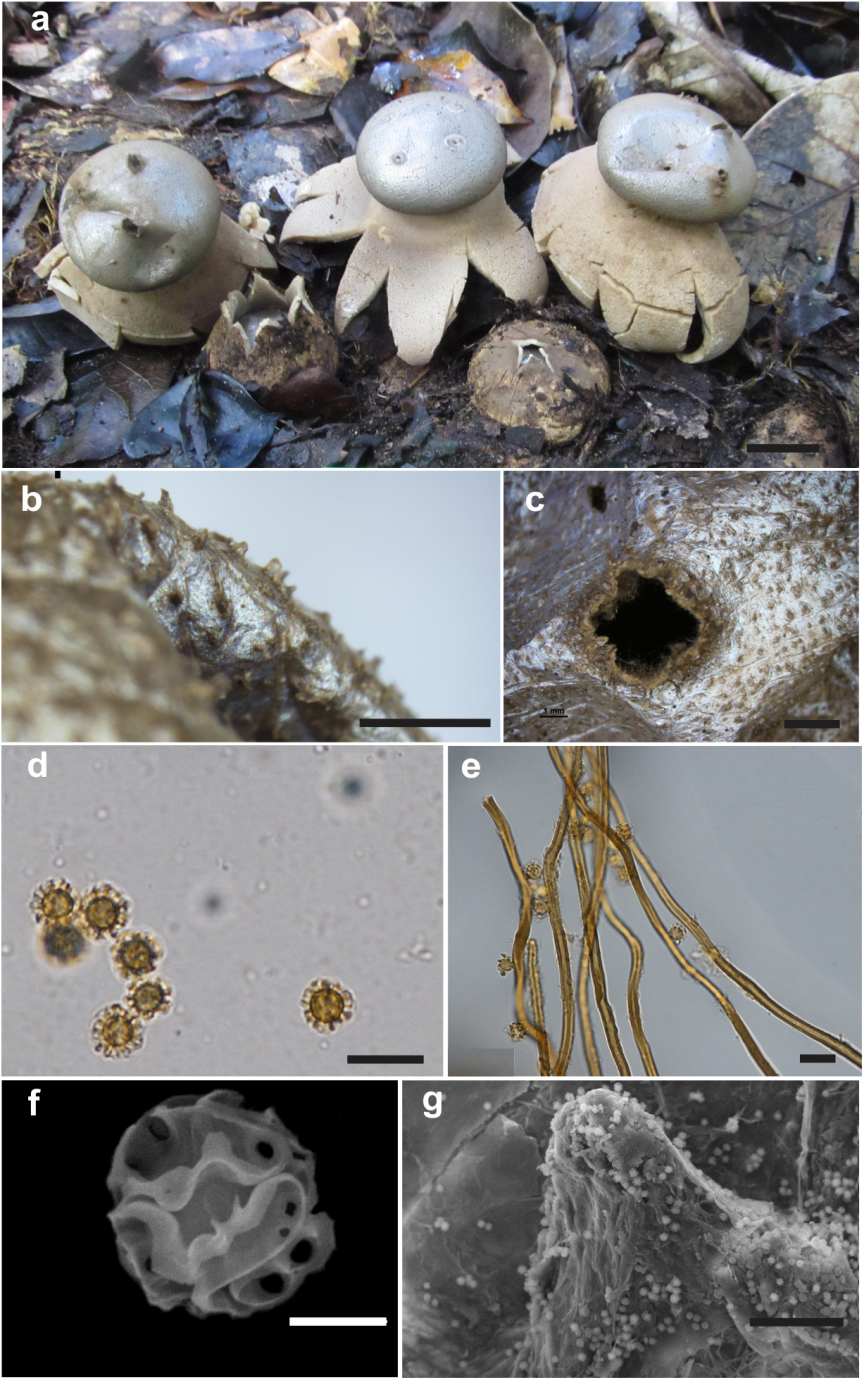
*Myriostoma calongei*. (a) Fresh expanded and unexpanded basidiomata *in situ* (UFRN-Fungos 2019, holotype), bar = 20 mm. (b) Endoperidial surface (UFRN-Fungos 386, paratype), bar = 1 mm. (c) Stoma (UFRN-Fungos 386, paratype), bar = 2 mm. (d) Basidiospores under LM (UFRN-Fungos 2020, isotype), bar = 10 μm. (e) Capillitium under LM (UFRN-Fungos 2019, holotype), bar = 10 μm. (f) Basidiospores under SEM, bar = 2 μm. (g) Endoperidial surface under SEM (UFRN-Fungos 2019, holotype), bar = 50 μm.

***Myriostoma calongei*** Baseia, J.O. Sousa, & M.P. Martín, sp. nov., Fig 3, Mycobank MB 818593.

Etymology. In honour of Prof. Francisco Diego Calonge, for his great contribution to the study of gasteroid fungi.

Holotype. Brazil, Rio Grande do Norte: Baía Formosa, RPPN Mata Estrela, 6°22’31.8”S 35°01’22.4”W, 61 msl, fruiting on leaf litter, under *Ficus* sp., 15 July 2012, leg. B.D.B. Silva & J.O. Sousa (UFRN-Fungos 2019!).

Diagnosis. *Myriostoma calongei* differs from other *Myriostoma* species mainly by the verrucose endoperidium, with prominent triangular processes (warts 0.13–0.28 mm high). It is closely related to *M*. *capillisporum*, but *M*. *calongei* has smaller basidiospores (5.9–8.7 μm diam) with less prominent ornamentation (1.0–2.3 μm high).

Description. Unexpanded basidiomata semi-hypogeous, globose to depressed globose, 22–30 mm × 18–37 mm, surface brown (6E4), papery, with longitudinal cracks, not encrusted to slightly encrusted with debris. Expanded basidiomata arched to saccate, 15–59 × 23–120 mm. Exoperidium splitting into 4–8 rays, arched to involute, rolling up under the endoperidial body, semi-hygroscopic to non- hygroscopic. Mycelial layer brown (6E4), dark brown (6F4) to greyish brown (6F3), papery, slightly encrusted to not encrusted with debris, peeling off in longitudinal cracks or in irregular patches. Fibrous layer brownish orange (5C3), white (5A1), orange white (5A2), greyish orange (5B3), coriaceous. Pseudoparenchymatous layer dark brown (6F4; 7F4), brown (6E4; 6E5), persistent or peeling off in irregular patches. Endoperidial body greyish brown (6D3), light brown (6D4) to orange grey (6B2), brownish grey (6D2), depressed globose to globose, 9–22 × 15–55 mm, surface slightly metallic and shiny, verrucose, warts 0.13–0.28 mm high. Multiple pedicels (5–13), 1.6–3.6 mm high, concolorous with the endoperidium, laterally compressed. Multiple stomata (3–11), fibrillose, scattered across the surface of the endoperidial body, slightly delimited, non-depressed on the endoperidium, lacerate with age, 2.5–3.8 mm diam. Gleba brown (6E5) to dark brown (6F3; 6F5).

Endoperidial surface with prominent triangular warts, 0.13–0.28 mm high under SEM. Mycelial layer composed of hyaline to brownish hyphae 2.5–5.1 μm diam, thin-walled (0.6–1.1 μm), non-incrusted, lumen not seen. Fibrous layer composed of hyaline sinuous hyphae 3.6–6.5 μm diam, thin-walled (0.4–1.0 μm), lumen not seen. Pseudoparenchymatous layer composed of hyaline to yellowish, thin to thick-walled hyphal cells, pyriform, subglobose to elongated, 20.4–41.1 × 10.5–32.8 μm. Eucapillitium of brownish hyphae 1.6–4.7 μm diam, thick-walled (0.3–0.9 μm), sinuous, encrusted, lumen seen. Basidiospores yellowish, subglobose, 5.9–8.7 × 5.6–7.6 μm [*x* = 6.9 ± 0.6 × 6.6 ± 0.5, Q_m_ = 1.06, n = 120], warts prominent (1.0–2.3 μm high) under LM; under SEM, the ornamentation is reticulate formed by confluent warts and ridges which are planar or curved when seen in face view.

Known distribution. South America (Brazil and Argentina).

Additional specimens examined. Argentina, Colón, Ubajay, El Palmar, S. Suaza, next to *Allophyllus edulis* and *Ligustrum lucidum*, 31 May 2012, leg. J. Maller & J.C. Zamora (MA-Fungi 83759, paratype). Brazil, Rio Grande do Norte, Baía Formosa, RPPN Mata Estrela, 6°22’32.1”S 35°01’21.6”W, 12 Jun. 2006, leg. B.D.B. Silva, J.O. Sousa & A.G. Leite (UFRN-Fungos 2020, isotype); Pernambuco, Buíque, Parque Estadual Vale do Catimbau, 8°30’22”S 37°19’23”W, fruiting on humid ground, 4 Aug. 2006, leg. J.F. Silva (UFRN-Fungos 386, paratype); Morro do Cachorro, 8°34’01”S 37°14’19”W, fruiting on ground under *Ziziphus* sp., 16 Apr. 2007, leg. T. Ottoni, (UFRN-Fungos 990, paratype).

Remarks. Specimens of this new species were identified in Sousa et al. [21] as *M*. *coliforme*. In Brazil, this species occurs in two vegetation types with quite different characteristics: Atlantic Rain Forest and “Caatinga”. The Atlantic Rain Forest is a “hotspot” of biodiversity comprising tropical forest formations, which extend along the east coast of the South American continent, while “Caatinga” is a vegetation type endemic to Brazil found in semiarid regions and specialized for life in a dry climate [51,52,53,54]. According to the specimen label, in Argentina this species was found next to endemic (*Allophyllus edulis*) and introduced (*Ligustrum lucidum*) plants.

**Fig 4:**
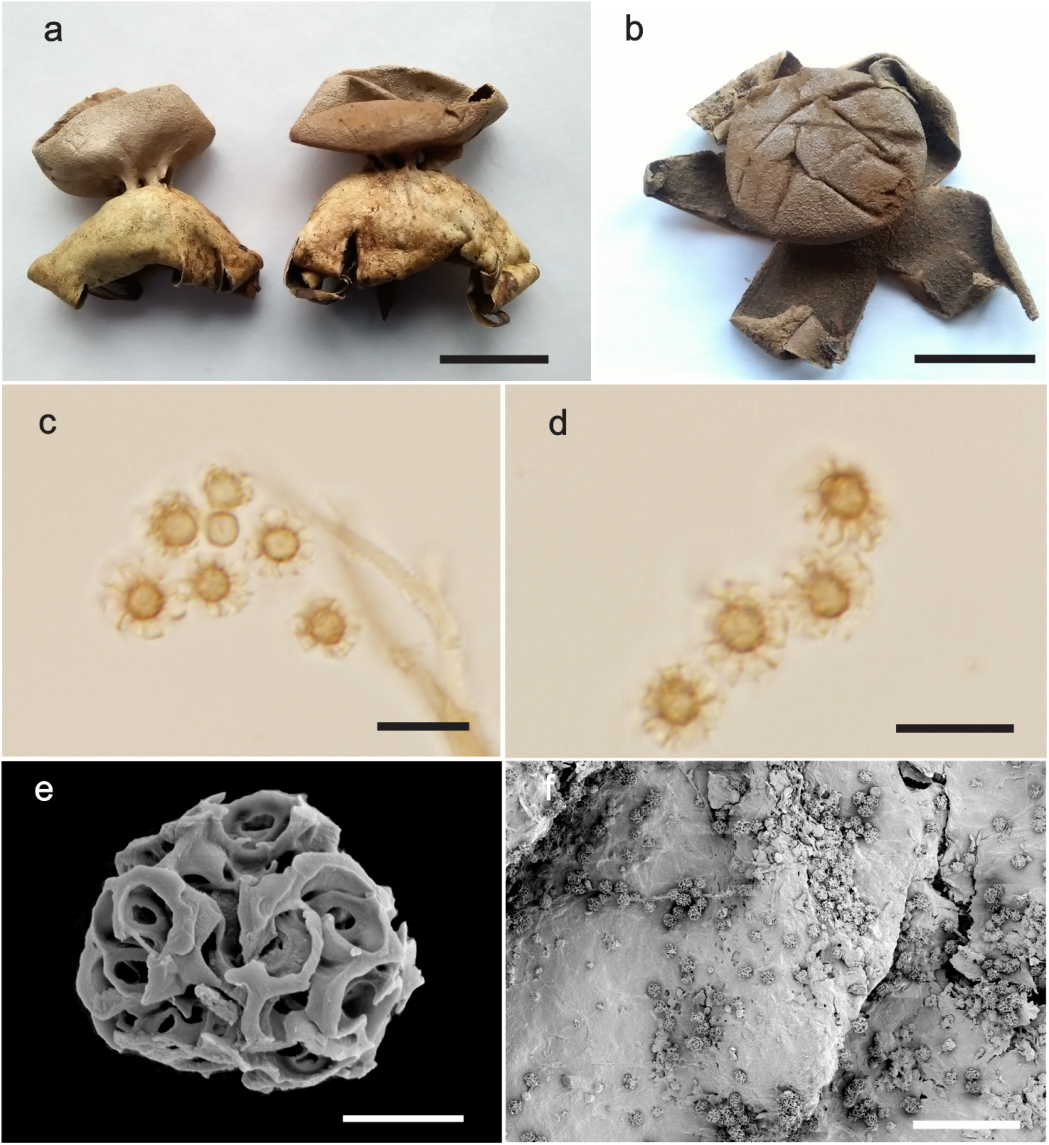
*Myriostoma capillisporum*. (a–b) Dried expanded basidiomata *ex situ* (KM205483 and K(M)205482, respectively), bar = 20 mm. (c–d) Basidiospores under LM (K(M)205483), bar = 10 μm. (e) Basidiospores under SEM (K(M)205483), bar = 2.5 μm. (f) Endoperidial surface under SEM (K(M)205483), bar = 50 μm.

***Myriostoma capillisporum*** (V.J. Staněk) L.M. Suz, A.M. Ainsw., Baseia & M.P. Martín, comb. & stat. nov., Fig 4, Mycobank MB 818616.

Etymology. Derived from Latin *capillu****s*,** meaning hair and referring to the prominent hair-like spore ornamentation.

Basionym. *Myriostoma coliforme* var. *capillisporum* V.J. Staněk, in Pilát (ed.) Flora ČSR B1 –Gasteromycetes: 402 (1958), MB 347330.

Type. South Africa, Cape Province, Belvidere, A.V. Duthie No. 31355 (Herb?).

Diagnosis. The basidiospore size, (7.0–)7.4–10.9 μm, and ornamentation comprising prominent warts under LM (2.9–6.6 μm high), which under SEM are formed by warts and ridges with confluent tips forming arcs and circles in face view, clearly distinguish this species from the other *Myriostoma* spp.

Etymology. Derived from Latin *capillus*, meaning hair and referring to the prominent hair-like spore ornamentation.

Expanded basidiomata arched 31–37 × 33–68 mm. Exoperidium splitting into 6–7 rays, arched to revolute, rolling up under the basidioma, non-hygroscopic. Pseudoparenchymatous layer ephemeral, absent in some basidiomata. Endoperidial body depressed globose, 11–12 × 26–35 mm (excluding pedicels), slightly verrucose, stalked. Multiple pedicels (6–7), 2.3–2.9 mm high, paler than or concolorous with the endoperidium. Multiple stomata (up to 4), fibrillose, scattered across the surface of the endoperidial body, lacerate with age. Endoperidial surface with irregular processes under SEM. Basidiospores light yellowish, subglobose, (7.0–)7.4–10.9 μm [x = 8.5 ± 0.8 n = 27], warts prominent (2.9–6.6 μm high) under LM; the ornamentation is reticulate under SEM, formed by warts and ridges with confluent tips, forming arcs and circles in face view.

Known distribution. South Africa.

Additional material studied. South Africa, Cape of Good Hope, pre 1885, Mac Owan (ex herb. M.C. Cooke), (K(M)205540, Kew Fungarium); Grahamstown, 1930s, leg. N.J.G. Smith, Smith 334, (K(M)205482, Kew Fungarium); Groot River, 1930s, leg. N.J.G. Smith, Smith 320, (K(M)205483, Kew Fungarium).

**Fig 5:**
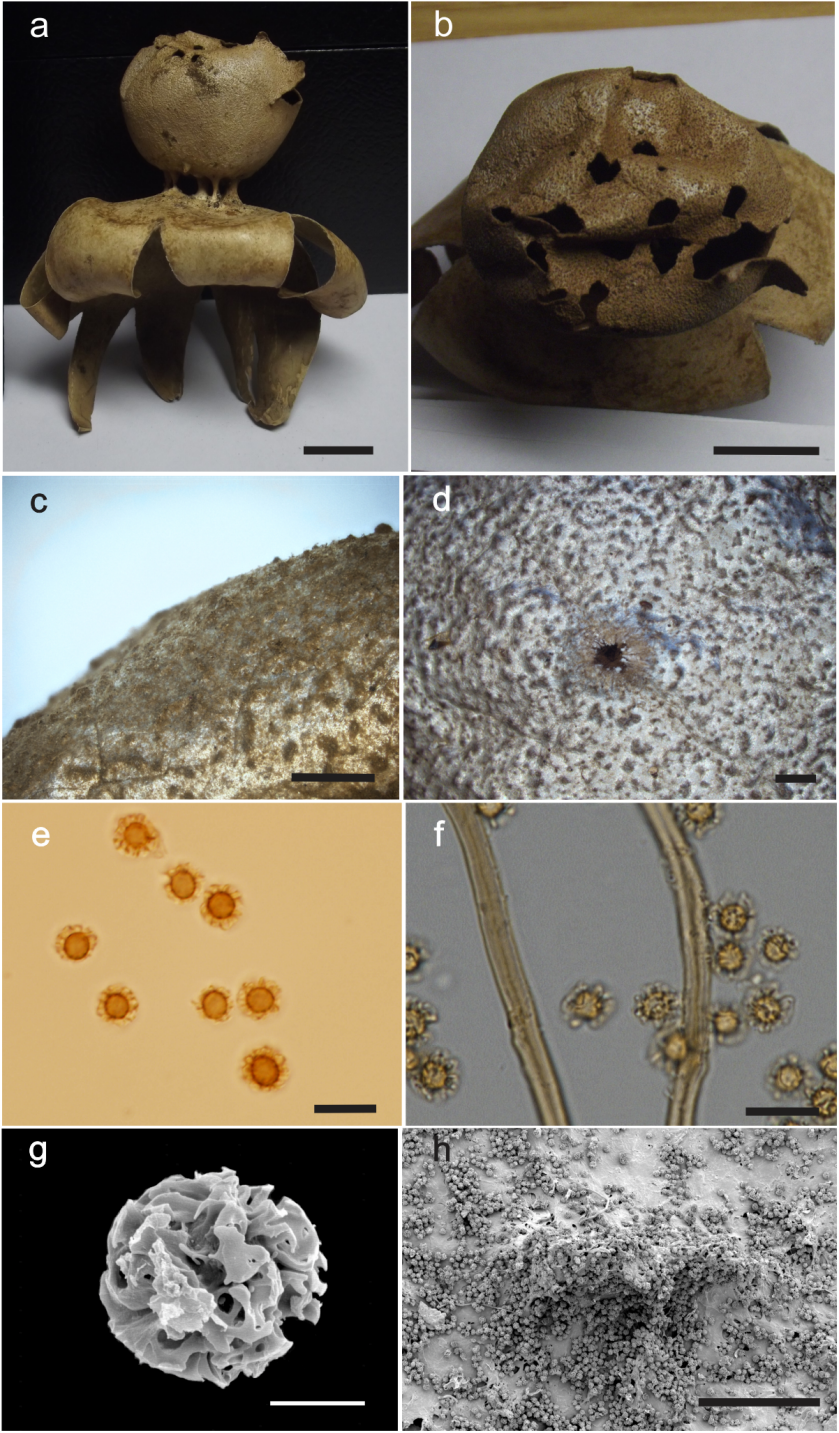
*Myriostoma coliforme*. **(**a–b) Dried expanded basidiomata *ex situ* (K(M)138625, epitype), bar 10 mm. (c) Endoperidial surface (PC0723885), bar = 1 mm. (d) Stoma (MJ8714), bar = 1 mm. (e) Basidiospores under LM (K(M)138625, epitype), bar = 10 μm. (f) Capillitium under LM (MJ8714), bar = 10 μm. (g) Basidiospores under SEM (K(M)138625, epitype), bar = 2.5 μm. (h). Endoperidial surface under SEM (K(M)138625, epitype), bar = 100 μm.

***Myriostoma coliforme*** (Dicks.) Corda 1842 [55], Fig 5, Mycobank MB 122233

Etymology. The specific epithet *coliforme* means colander- or strainer-like, because “colum” means strainer in Latin, referring to the multistomatous endoperidium.

**Fig 6:**
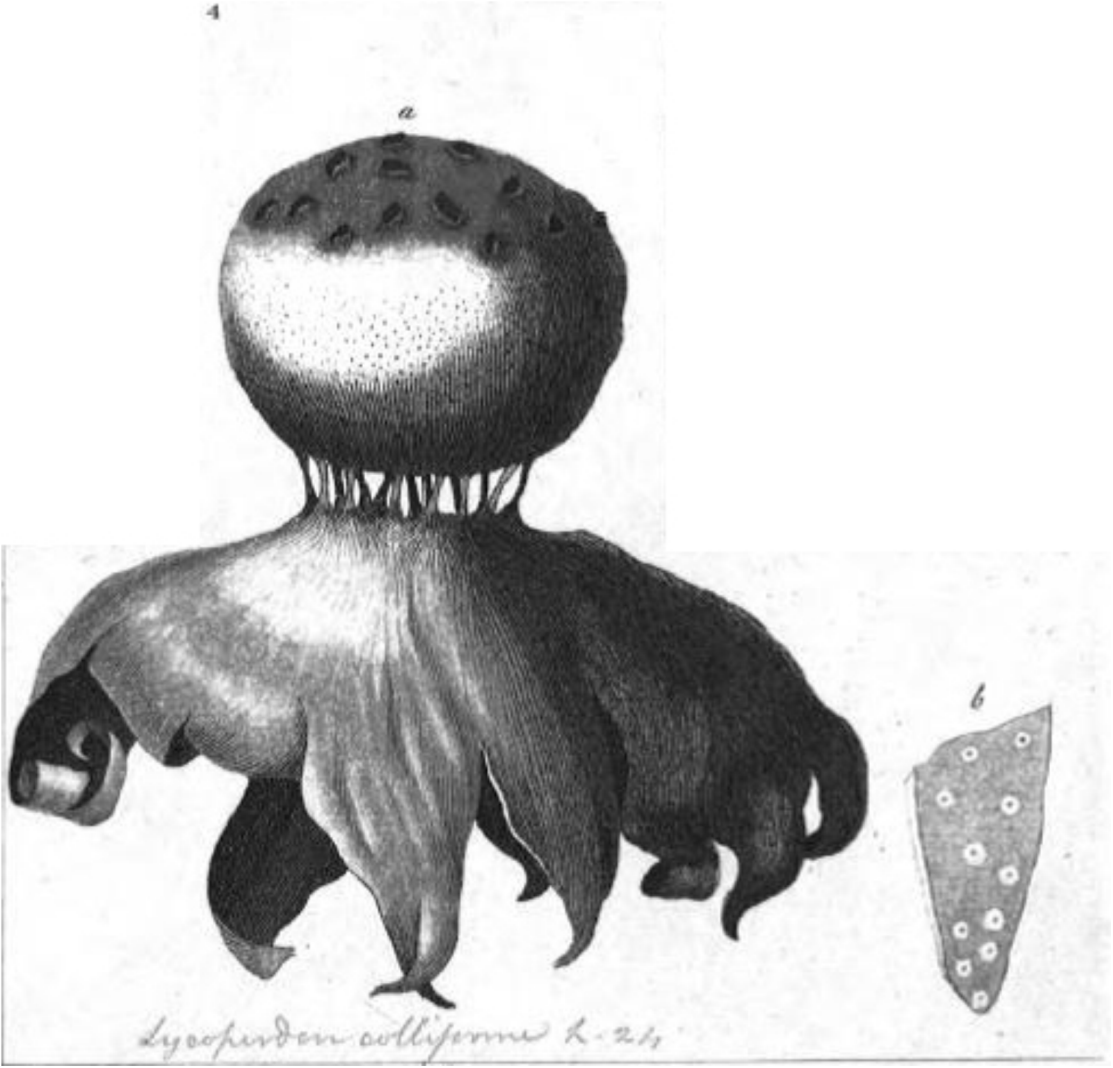
Dickson’s illustration of *Myriostoma coliforme* (lectotype) published in 1785 as (Tab. III: Fig 4).

Lectotype (designated here). Dickson’s illustration [as *Lycoperdon colliforme*], in Dickson (1785) Fasc. pl. crypt. brit. (London) 1:24 (Tab. III: Fig 4), reproduced as Fig 6, IF 552745.

Epitype (designated here). United Kingdom: England. East Suffolk (vice county 25), north of Ipswich, Nat. Grid Ref. TM15, fruiting in sandy soil, 19 February 2006, leg. C. Povey (K(M)138625!), IF 552746.

Supported lectotypification. See above.

Basionym. *Lycoperdon coliforme* Dicks., Fasciculus plantarum cryptogamicarum Britanniae 1:24 (1785), MB 228521.

Diagnosis. *Myriostoma coliforme* is closely related to *M*. *areolatum*, but clearly distinguished by the presence of flattened stomata, whereas in *M*. *areolatum* the stomata are areolate and tubular.

Description. Expanded basidioma arched 38–135 mm wide. Exoperidium splitting into 6–11 rays arched, involute to horizontal, non-hygroscopic. Mycelial layer greyish yellow (3D4), papery, encrusted with debris, squamous or slightly peeling in longitudinal cracks in some basidiomata. Fibrous layer pastel yellow (1A4) to pale yellow (2A2), papery. Pseudoparenchymatous layer greyish yellow (3C4; 4B4) to light brown (5C6), persistent or peeling in some basidiomata. Endoperidial body pale yellow (2A3), blond (4C4) to yellowish brown (5D4), depressed globose to globose, 18–40 × 24–50 mm (excluding pedicels), surface wrinkled to slightly verrucose (warts < 0.1 mm high with rounded tips), stalked. Multiple pedicels (3–9), 3–10 mm high, concolorous with the endoperidial body. Multiple stomata (6–24), >30 visible in some Swedish specimens illustrated in Sunhede (1989), fibrillose, flattened, non-delimited, lacerate with age, 1.5–5 mm diam. Gleba light brown (5E5) to brown (6E7).

Endoperidial surface comprised of small processes under SEM. Eucapillitial hyphae brownish 3.3–4.0 μm diam, thick-walled (0.9–1.5 μm), sinuous, non-encrusted, lumen seen. Basidiospores yellowish, subglobose 6.1–8.0 μm [x = 7.0±0.4 n = 60], warts 1.2–1.6 μm high under LM; under SEM, the ornamentation is reticulate, formed by warts and ridges with planar or slightly curved tips in surface view.

Known distribution. Europe, North America and Oceania (Hawai’i).

Additional material studied: Channel Islands, Jersey, Les Vaux Cuissin, fruiting on sandy soil, 23 May 1999, leg. B.M. Spooner (K(M)61641, Kew Fungarium); St Ouen's, fruiting on old sand dunes, Feb. 1996, leg. N. Armstrong (K(M)37233, Kew Fungarium). France, 64 Pyrénées-Atlantiques, Région de Nay (Basses Pyrénées), 1964, leg. J. Beller (PC 0723885). Hungary, Bács-Kiskun, Ladánybene, Felsolajos, 8 Sep. 2003, leg. L. Nagy & M. Jeppson (personal herbarium MJ8714; ITS and LSU nrDNA Acc. Number KC582020). Portugal, Leiria, Mato das Acacias, 2 Apr. 1983, leg. L. Freire & M. Castro (MA-Fungi 31316); Maderia, Jardines de la Universidad, under *Cupressus* sp., 8 Aug. 2007, leg. M. Sequeira (MA-Fungi 75818). Russia, Rostov region, Sholokhovsky District, Schebunyaevsky village, fruiting on pasture soil, 20 July 2004, leg. Y. Rebriev 1090 (K(M)154620, Kew Fungarium). Spain, Jaén, Andújar, Las Viñas, under *Eucalyptus* sp. 18 Nov. 2004, leg. F. Jiménez (MA-Fungi 60898); Balearic Islands, Menorca, Maó, Sant Antoni, under *Quercus ilex*, 27 Nov. 1998, leg. B. Mateo (MA-Fungi 40288). United Kingdom: England, East Suffolk, near Harleston, fruiting on sandy soil, 30 July 2010, leg. N. Mahler (K(M)166470, Kew Fungarium); idem, 18 Nov. 2014, leg. N. Mahler (K(M)195584, Kew Fungarium); England, West Norfolk, Hillington, fruiting on soil, Oct. 1880, leg. P. Hebgin (via Lady Ffolkes) (K(M)81165, Kew Fungarium).

Sequences retrieved from GenBank (specimens not studied morphologically). USA, Hawai‘i, TKG-GE-50801 (TNS Herbarium; ITS sequence Acc. Number JN845203; LSU sequence Acc. Number JN845328).

Remarks. Although we have not analysed DNA from specimens from the USA, other than those from Hawai‘i, this species is widespread in North America. Descriptions of North American material provided in Coker and Couch [56] and Bates [15] accord with the lectotypification proposed here. Specimens were recorded under desert hackberry (*Celtis pallida*), juniper (*Juniperus* spp.), mesquite (*Prosopis* spp.) or cactus species [15]. The presence of *M*. *coliforme* on Hawai‘i Island was also recorded in Smith and Ponce de León [57] and Gilbertson et al. [58], under *Sophora chrysophylla*, an endemic Fabaceae; moreover, Hemmes and Desjardin [18] collected numerous specimens in Manuka Wayside Park (Hawai‘i Island) under several introduced and endemic plants.

Tejera et al. [59] provided descriptions of specimens identified as *M*. *coliforme* from the Canary Islands that are also in accordance with the lectotypified concept. Esqueda-Valle et al. [19,60] recorded *M*. *coliforme* in Mexico (Sonora Desert) under *Prosopis* sp.; however, there are no descriptions to confirm that these authors are referring to the lectotypified concept. In South America, there are several records of *M*. *coliforme*, mostly from southeast Brazil [61–62] and from areas of “Caatinga” vegetation in northeast Brazil: under *Spondias tuberos* [63] and under *Ficus* sp. [21, 64], With the exception of the specimens in Sousa et al. [21], the South American collections were not subjected to DNA sequence analysis, but based on morphological characters alone they should be assigned to *M*. *calongei*.

## Discussion

**Fig 7:**
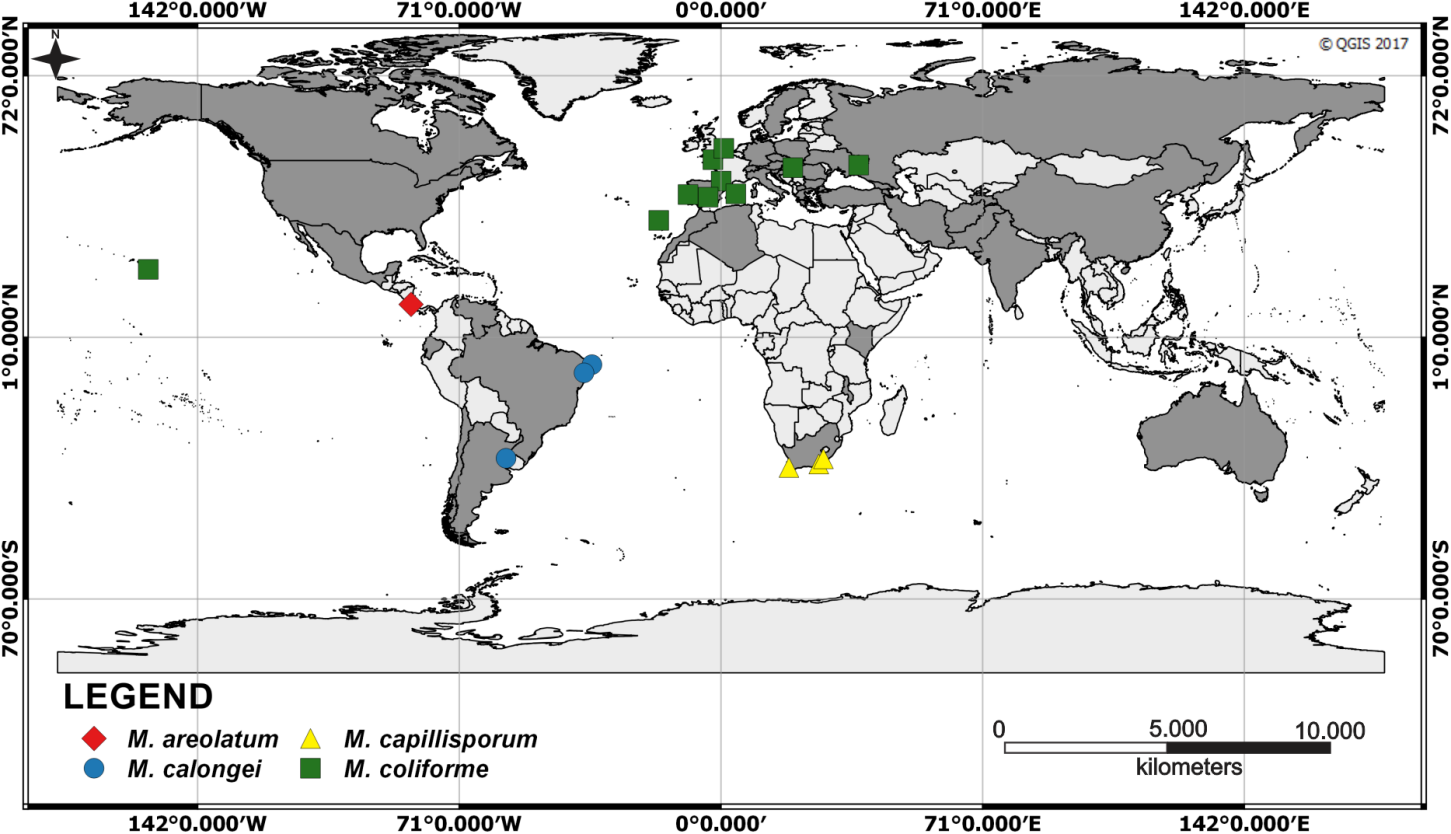
Distribution map of the *Myriostoma* specimens included in the phylogenetic analyses of this study (geometric figures in colour). This figure was made using the software Quantum Gis 2.8 (QGIS), a free and open source geographic information system (https://www.qgis.org/es/site/about/index.html). The dark grey areas correspond to the distribution of *Myriostoma coliforme* s.l. according to http://iucn.ekoo.se/iucn/species_view/122233/ (accessed on 12.01.2017) and Fraiture & Otto [22].

Since the nineteenth century, the genus *Myriostoma* has been regarded as monotypic. Pegler et al. [14] indicated that *M*. *coliforme* is widespread in north temperate and subtropical regions. However, our study reveals that the name *M*. *coliforme* has been applied to at least four members of a species complex each of which is well characterized by a combination of morphological characters, of which the stomata, endoperidial surface and spore size and ornamentation are the most important. Consequently, the distribution of *M*. *coliforme* in the original sense has been overestimated (IUCN webpage: http://iucn.ekoo.se/iucn/species_view/122233/; [22]; Fig 7). Although further worldwide sampling is clearly required, current DNA-based evidence supports a European and North American range for *M*. *coliforme*.

A lack of knowledge about dispersal mechanisms coupled with insufficient molecular data on Neotropical fungi have resulted in speculative interpretations of their biogeographic distribution, especially for saprotrophic taxa such as *Myriostoma* [20,65]. Recent studies demonstrate that fungal species with a cosmopolitan distribution are the exception [66,67]. In general, most names applied to species with an apparent worldwide distribution probably represent species complexes rather than good species [68]. Based on the conclusion of Kasuya et al. [20] regarding the earthstar *Geastrum triplex* Jungh., which has a similar, bellows-like, spore dispersal mechanism, *Myriostoma* dispersal capacity is not expected to be very effective over long distances.

This work opens new perspectives on this striking genus through the application of integrative taxonomy using a combined molecular and morphological approach. Indeed, the production of revised distribution maps of *Myriostoma* species is an essential prerequisite for ecological studies and robust and reliable IUCN-compliant conservation assessments.

## References

[1] Blackwell M. The Fungi: 1, 2, 3 … 5.1 million species? Am J Bot. 2011; 98(3): 426–438. 21613136 10.3732/ajb.1000298

[2] Wilson AW, Binder M, Hibbett DS. Effects of gasteroid fruiting body morphology on diversification rates in three independent clades of fungi estimated using binary state speciation and extinction analysis. Evolution. 2011; 65(5): 1305–1322. doi: 10.1111/j.1558-5646.2010.01214.x 21166793 10.1111/j.1558-5646.2010.01214.x

[3] Hawksworth DL. Global species numbers of fungi: are tropical studies and molecular approaches contributing to a more robust estimate? Biodivers Conserv. 2012; 21(9): 2425–2433.

[4] Frøslev TG, Jeppesen TS, Laessøe T, Kjøller R. Molecular phylogenetics and delimitation of species in *Cortinarius* section Calochroi (Basidiomycota, Agaricales) in Europe. Mol Phylogenet Evol. 2007; 44: 217–227. doi: 10.1016/j.ympev.2006.11.013 17197201 10.1016/j.ympev.2006.11.013

[5] Vizzini A, Della Magiora M, Tolaini F, Ercole E. A new cryptic species in the genus *Tubariomyces* (Inocybaceae, Agaricales). Mycol Progress. 2013; 12: 375–381.

[6] Beker HJ, Eberhardt U, Vesterholt J. Hebeloma (Fr.) P. Kumm. Fungi Europaei 14. Edizione Tecnografica, Lomazzo; 2016.

[7] Stielow B, Bratek Z, Orczán AKI, Rudnoy S, Hensel G, Hoffmann P et al Species delimitation in taxonomically difficult Fungi: The Case of Hymenogaster. PLoS ONE 2011; 6(1): e15614 doi: 10.1371/journal.pone.0015614 21311589 10.1371/journal.pone.0015614 PMC3027480

[8] Schoch CL, Seifert KA, Huhndorf S, Robert V. Spouge JL, Levesque CA et al Nuclear ribosomal internal transcribed spacer (ITS) region as a universal barcode marker for Fungi. P Natl Acad Sci USA. 2012; 109(16): 6241–6246. 10.1073/pnas.1117018109 PMC3341068 22454494

[9] Phosri C, Martín MP, Suwannasai N, Sihanonth P, Watling R. *Pisolithus*: a new species from southeast Asia and a new combination. Mycotaxon. 2012; 120: 195–208.

[10] Phosri C, Martín MP, Watling R. *Astraeus*: hidden dimensions. IMA Fungus. 2013; 4(2): 347–356. doi: 10.5598/imafungus.2013.04.02.13 24563840 10.5598/imafungus.2013.04.02.13 PMC3905946

[11] Phosri C, Watling R, Suwannasai N, Wilson A, Martín MP. A new representative of star-shaped Fungi: *Astraeus sirindhorniae* sp. nov. from Thailand. PloS ONE. 2014; 9(5): e71160 doi: 10.1371/journal.pone.0071160 24806455 10.1371/journal.pone.0071160 PMC4012956

[12] Martín MP, Durán F, Phorsi C, Watling R. A new species of *Pisolithus* from Spain. Mycotaxon. 2013; 124: 149–154.

[13] Sunhede S. Geastraceae (Basidiomycotina): Morphology, ecology and systematics with special emphasis on the North European species (Synopsis Fungorum, vol. 1). Fungiflora, Oslo; 1989.

[14] Pegler DN, Læssøe T, Spooner BM. British puffballs, earthstars, and stinkhorns. Royal Botanic Gardens, Kew; 1995.

[15] Bates ST. Arizona members of the Geastraceae and Lycoperdaceae (Basidiomycota, Fungi). Master’s Thesis, Arizona State University; 2004.

[16] Calonge FD, Mata M. Adiciones y correcciones al catálogo de Gasteromycetes de Costa Rica. Bol Soc Micol Madrid. 2006; 30: 111–119.

[17] Dios MM, Alberto E, Moreno G. Catálogo de hongos gasteroides (Basidiomycota) de Catamarca, Argentina. Bol Soc Argent Bot. 2011; 46(1–2): 5–11. http://www.scielo.org.ar/pdf/bsab/v46n1-2/v46n1-2a01.pdf

[18] Hemmes DE, Desjardin DE. Earthstars (*Geastrum*, *Myriostoma*) of the Hawaiian Islands including two new species, *Geastrum litchiforme* and *Geastrum reticulatum*. Pac Sci. 2011; 65: 477–496.

[19] Esqueda-Valle M, Sánchez A, Riversa M, Conrado ML, Lizárraga M, Valenzuela R. Primeros registros de hongos gasteroides en la Reserva Forestal Nacional y Refugio de Fauna Silvestre Ajos-Bavispe, Sonora, México. Rev Mex Mic. 2009; 30: 19–29. http://www.scielo.org.mx/pdf/rmm/v30/v30a3.pdf

[20] Kasuya T, Hosaka K, Uno K, Kakishima M. Phylogenetic placement of *Geastrum melanocephalum* and polyphyly of *Geastrum triplex*. Mycoscience. 2012; 53(6): 411–426.

[21] Sousa JO, Silva BDB, Alfredo DS, Baseia IG. New records of Geastraceae (Basidiomycota: Phallomycetidae) from Atlantic Rainforest remnants and relicts of Northeastern Brazil. Darwiniana, Nueva Serie. 2014; 2(2): 207–221.

[22] Fraiture A, Otto P. Distribution, ecology and status of 51 macromycetes in Europe (Scripta Botanica Belgica, vol. 53. Botanic Garden Meise, Meise; 2015.

[23] Jeppson M, Nilsson RH, Larsson E. European earthstars in Geastraceae (Geastrales, Phallomycetidae)–a systematic approach using morphology and molecular sequence data. Syst Biodivers. 2013; 11 (4): 437–465.

[24] Pilát A. Flora ČSR B–1, Gasteromycetes, Houby-Břichatky. Československá Akademie Vĕd, Prague; 1958.

[25] Kornerup A, Wanscher JE. Methuen handbook of colour, 3rd ed. Methuen, London; 1978.

[26] Gardes M, Bruns TD. ITS primers with enhanced specificity for Basidiomycetes applications to the identification of mycorrhizae and rusts. Mol Ecol. 1993; 1: 113−118. 10.1111/j.1365-294x.1993.tb00005.x 8180733

[27] White TJ, Bruns T, Taylor J. Amplification and direct sequencing of fungal ribosomal RNA genes for phylogenetics In: Innes MA, Gelfand DH, Sninsky JJ, White TJ. PCR protocols. A guide to methods and applications. Academic Press, Inc., California, San Diego 1990; pp. 315−322. https://nature.berkeley.edu/brunslab/papers/white1990.pdf

[28] Rehner SA, Samuels GJ. Taxonomy and phylogeny of *Gliocladium* analyzed from nuclear large subunit ribosomal DNA sequences. Mycol Res. 1994; 98: 625–634.

[29] Vilgalys R, Hester M. Rapid genetic identification and mapping of enzymatically amplified ribosomal DNA from several *Cryptococcus* species. J Bacteriol. 1990; 172: 4238–4246. 2376561 10.1128/jb.172.8.4238-4246.1990 PMC213247

[30] Martín MP, Winka K. Alternative methods of extracting and amplifying DNA from lichens. Lichenologist. 2000; 32(2): 189–196.

[31] Dentinger BTM, Margaritescu S, Moncalvo JM. Rapid and reliable high-throughput methods of DNA extraction for use in barcoding and molecular systematics of mushrooms. Mol Ecol Resources. 2010; 10: 628–633. 10.1111/j.1755-0998.2009.02825.x 21565067

[32] Dentinger BTM, Suz LM. What’s for dinner? Undescribed species of porcini in a commercial packet. PeerJ. 2014; 2: e570 doi: 10.7717/peerj.570 25279259 10.7717/peerj.570 PMC4179395

[33] Altschul SF, Madden TL, Schäffer AA, Zhan J, Zhan GZ, Millar W et al Gapped BLAST and PSI-BLAST: a new generation of protein database search programs. Nucleic Acid Res. 1997; 25: 3389–3402. 9254694 10.1093/nar/25.17.3389 PMC146917

[34] Rambaut A. Se-Al: sequences alignment editor v2.0a11. Institute of Evolutionary Biology, University of Edinburgh; 2002. http://tree.bio.ed.ac.uk/software/

[35] Cochrane G, Karsch-Mizrachi I, Nakamura Y. The International Nucleotide Sequence Database Collaboration. Nucleic Acid Res. 2011; 39:D15–18. doi: 10.1093/nar/gkq1150 21106499 10.1093/nar/gkq1150 PMC3013722

[36] Zamora JC, Calonge FD, Hosaka K, Martín MP. Systematics of the genus *Geastrum* (Fungi: Basidiomycota) revisited. Taxon. 2014; 63(3): 477–497.

[37] Baum DA, Sytsma K, Hoch P. A molecular phylogenetic analysis of *Epilobium* based on sequences of nuclear ribosomal DNA. Syst Bot. 1994; 19: 363–388. http://www.jstor.org/stable/2419763

[38] Swofford DL. PAUP*. Phylogenetic analysis using parsimony (*and other methods) Version 4. Sinauer Associates, Sunderland, Massachusetts; 2003.

[39] Felsenstein J. Confidence limits on phylogenies: an approach using the bootstrap. Evolution. 1985; 39: 783–791. http://www.jstor.org/stable/2408678 28561359 10.1111/j.1558-5646.1985.tb00420.x

[40] Mort ME, Soltis PS, Soltis DE, Macry MK. Comparison of three methods for estimating internal support on phylogenetic trees. Syst Biol. 2000; 49: 60–171. 10.1080/10635150050207456 12116478

[41] Kluge AG, Farris JS. Quantitative phyletics and the evolution of anurans. Syst Zool. 1969; 18: 1–32.

[42] Farris JS. The retention index and the rescaled consistency index. Cladistics. 1989; 5: 417–419. 10.1111/j.1096-0031.1989.tb00573.x 34933481

[43] Stamatakis A. RAxML Version 8: A tool for phylogenetic analysis and post-analysis of large phylogenies. Bioinformatics. 2014; 30(9): 1312–1313. doi: 10.1093/bioinformatics/btu033 24451623 10.1093/bioinformatics/btu033 PMC3998144

[44] Larget B, Simon DL. Markov chain Monte Carlo algorithms for the Bayesian analysis of phylogenetic trees. Mol Biol Evol. 1999; 16: 750–759. http://pages.stat.wisc.edu/~larget/phylogeny/larget-simon-MBE-1999.pdf

[45] Huelsenbeck JP, Ronquist F, Nielsen R, Bollback JP. Bayesian inference of phylogeny and its impact on evolutionary biology. Science. 2001; 294: 2310–2314. doi: 10.1126/science.1065889 11743192 10.1126/science.1065889

[46] Ronquist F, Teslenko M, Van der Mark P, Ayres DL. Darling A, Höhna S et al MrBayes 3.2: efficient Bayesian phylogenetic inference and model choice across a large model space. Syst Biol. 2012; 61:539–542. doi: 10.1093/sysbio/sys029 22357727 10.1093/sysbio/sys029 PMC3329765

[47] Rodriguez F, Oliver JF, Martín A, Medina JR. The general stochastic model of nucleotide substitution. Journal of Theoretical Biology. 1990; 142: 485–501; 2338834 10.1016/s0022-5193(05)80104-3

[48] Lutzoni F, Kauff F, Cox CJ, McLaughlin D, Celio G, Dentinger B et al Assembling the fungal tree of Life: Progress, classification, and evolution of subcellular traits. Am J Bot. 2004; 91(10): 1446–1480. doi: 10.3732/ajb.91.10.1446 21652303 10.3732/ajb.91.10.1446

[49] Persoon CH. Neuer versuch; einer systematischen eintheilung der schwämme. Neues Mag Bot. 1794; 1: 63–124.

[50] Dickson J. Fasciculus plantarum cryptogamicarum Britanniae. London; 1785.

[51] Myers N, Mittermeier RA, Mittermeier CG, Fonseca GAB, Kent J. Biodiversity hotspots for conservation priorities. Nature. 2000; 403: 853–858. doi: 10.1038/35002501 10706275 10.1038/35002501

[52] Prado D. As Caatingas da América do Sul. In: Leal I, Tabarelli M, Silva JMC. Ecologia e conservação da caatinga. Ed. Universitária, UFPE, Recife. 2003; pp. 3–73.

[53] Leal IR, Silva JMC, Tabarelli M, Lancher TE Jr.. Mudando o curso da conservação da biodiversidade na caatinga do Nordeste do Brasil. Megadiversidade. 2005; 1(1): 139–146. https://portais.ufg.br/up/160/o/19_Leal_et_al.pdf

[54] Tabarelli M, Pinto LP. Silva JCM, Hirota M, Bedê L. Challenges and opportunities for biodiversity conservation in the Brazilian Atlantic Forest. Conserv Biol. 2005; 19: 695–700.

[55] Corda AKJ. Anleitung zum studium der mykologie. F. Ehrlich, Prague; 1842.

[56] Coker WC, Couch JN. The Gasteromycetes of the Eastern United States and Canada. University of North Carolina Press, Chapel Hill; 1928.

[57] Smith CW, Ponce de León P. Hawaiian gasteroid fungi. Mycologia. 1982; 74: 712–717.

[58] Gilbertson RL, Desjardin DE, Rogers JD, Hemmes DE. Fungi from the mamane-naio vegetation zone of Hawai‘i. Fungal Divers. 2001; 6: 35–69.

[59] Tejera EB, Baudet AB, Rodríguez-Armas JL. Gasteromycetes of the Canary Islands. Some noteworthy new records. Mycotaxon. 1998; 67: 439–453.

[60] Esqueda-Valle M, Perez-Silva E, Herrera T, Martín FS, Santos-Gusmán R. Macromicetos de Selva Baja Caducifolia, I: Álamos, Sonora, México. Rev Mex de Mic. 1999; 15: 73–78.

[61] Rick J. Basidiomycetes Eubasidii no Rio Grande do Sul. Brasília. Iheringia. 1961; 9: 451–480.

[62] Homrich MA. Nota sobre *Myriostoma coliforme* Desvaux (Lycoperdaceae). Iheringia. 1973; 18: 80–89.

[63] Baseia IG, Galvão TCO. Some interesting Gasteromycetes (Basidiomycota) in dry areas from Northeastern Brazil. Acta Bot Brasil. 2002; 16: 1–8.

[64] Leite AG, Baseia IG. Novos registros de Geastraceae Corda para o Nordeste Brasileiro. Sitientibus Ser Ci Biol. 2007; 7: 178–183.

[65] Yang ZL. Molecular techniques revolutionize knowledge of basidiomycete evolution. Fungal Divers. 2011; 50: 47–58.

[66] Salgado-Salazar C, Rossman AY, Chaverri P. Not as ubiquitous as we thought: Taxonomic crypsis, hidden diversity and cryptic speciation in the cosmopolitan fungus *Thelonectria discophora* (Nectriaceae, Hypocreales, Ascomycota). PLoS ONE. 2013; 8(10): e76737 doi: 10.1371/journal.pone.0076737 24204665 10.1371/journal.pone.0076737 PMC3799981

[67] Peay KG, Kennedy PG, Talbot JM. Dimensions of biodiversity in the Earth mycobiome. Nat Rev Microbiol. 2016; 14: 434–447. doi: 10.1038/nrmicro.2016.59 27296482 10.1038/nrmicro.2016.59

[68] Lumbsch HT, Buchanan PK, May TW, Mueller GM. Phylogeography and biogeography of Fungi. Mycol Res. 2008; 112: 423–424. doi: 10.1016/j.mycres.2008.02.002 18346884 10.1016/j.mycres.2008.02.002

